# A Japanese adult and two girls with NEDMIAL caused by de novo missense variants in *DHX30*

**DOI:** 10.1038/s41439-021-00155-9

**Published:** 2021-06-18

**Authors:** Kimiko Ueda, Atsushi Araki, Atsushi Fujita, Naomichi Matsumoto, Tomoko Uehara, Hisato Suzuki, Toshiki Takenouchi, Kenjiro Kosaki, Nobuhiko Okamoto

**Affiliations:** 1 Department of Medical Genetics, Osaka Women’s and Children’s Hospital, Izumi, Osaka, Japan; 2 Nakano Children’s Hospital, Osaka, Japan; 3 Department of Human Genetics, Yokohama City University Graduate School of Medicine, Yokohama, Japan; 4 Center for Medical Genetics, Keio University School of Medicine, Tokyo, Japan; 5 Department of Pediatrics, Central Hospital, Aichi Developmental Disability Center, Aichi, Japan; 6 Department of Pediatrics, Keio University Hospital, Tokyo, Japan

**Keywords:** Clinical genetics, Medical research

## Abstract

Lessel et al. reported a novel neurodevelopmental disorder with severe motor impairment and absent language (NEDMIAL) in 12 individuals and identified six different de novo heterozygous missense variants in *DHX30*. The other clinical features included muscular hypotonia, feeding difficulties, brain anomalies, autistic features, sleep disturbances, and joint hypermobility. We report a Japanese adult with a novel missense variant and two girls with de novo missense variants in *DHX30*.

DHX30 is a member of the DEAH-box family of RNA helicases. *DHX30* is located on chromosome 3p21.31, and it encodes an ATP-dependent RNA helicase. The homozygous deletion of *DHX30* leads to early embryonic lethality and causes early developmental defects in the central nervous system in mice^1^. Lessel et al. reported a novel neurodevelopmental disorder with severe motor impairment and absent language (NEDMIAL; MIM #617804) in 12 individuals and identified six different de novo heterozygous missense variants in the *DHX30* gene^2^. Recently, two brothers with the same de novo missense variation in *DHX30* by gonadal mosaicism were reported^3^.

NEDMIAL is characterized by severely delayed psychomotor development, which causes an autosomal dominant disorder. The affected patients show muscular hypotonia, feeding difficulties, ataxic gait, or an inability to walk. Their cognitive development is severely impaired. Speech development is minimal or absent, and severe intellectual disability is a constant feature. It is also characterized by behavioral abnormalities, such as autistic features, low frustration tolerance, hand-flapping, and stereotypies. Additional common features may include sleep disorders, nonspecific dysmorphic facial features, and joint hypermobility.

We report herein a Japanese adult and two girls with de novo missense *DHX30* variants.

Patient 1 was a 22-year-old male who was the first child of healthy and nonconsanguineous Japanese parents. His birth weight was 2864 g (−1.3 standard deviations (SD)), length was 49 cm (−0.7 SD), and head circumference was 32 cm (−1.5 SD) at 41 weeks of gestation. He showed severe global developmental delay from the early infantile period. He was referred to the city hospital at 2 years of age, and a magnetic resonance imaging (MRI) brain scan indicated brain atrophy. Complex partial seizures were noted for the first time at age 3 and did not occur again subsequently, although electroencephalography revealed multifocal epileptic spikes. Puberty was delayed, and changes in his voice occurred after he was 20 years old. He gradually developed a Rett syndrome-like presentation. His karyotype by G-banding analysis was 46, XY. Rett syndrome and Angelman syndrome were excluded by molecular analysis. He was referred to our center for detailed examination. His height was 149 cm (−3.3 SD), weight was 33 kg (−3.5 SD), and head circumference was 49.3 cm (−0.6 SD) at 22 years of age. He was bedridden and could still not crawl or stand, although he could sit at the age of 3. He had scoliosis and bilateral hip dislocations. He also showed periodic hyperventilation, frequent bouts of laughing, and sleep disturbance. He showed all emotions without words and could not understand simple commands. He needed careful supervision and help in all of his daily activities.

Patient 2 was a 7-year-old girl who was the first child of healthy and nonconsanguineous Japanese parents. Her birth weight was 3258 g (−1.2 SD), length was 48 cm (+0.3 SD), and head circumference was 33.5 cm (−0.2 SD) at 41 weeks of gestation. She was hypotonic, and motor development was delayed. She could roll over at 12 months of age, crawl at 3 years of age, and sit at 4 years of age. Her expressive language was severely delayed. She gradually developed a Rett syndrome-like presentation. However, no variants or deletions were found in methyl-CpG-binding protein 2 (*MECP2*)^4^. She did not experience epileptic seizures. Physical examination revealed dysmorphic features, including thick low-set ears, long eyelashes, interdental space, a high arched palate, and micrognathia (Fig. 1a). Her hands and feet were small (Fig. 1b). Her height was 103 cm (−3.2 SD), weight was 14 kg (−2.4 SD), and head circumference was 47.1 cm (−3.1 SD). She could not stand or speak at 7 years of age. Brain MRI showed a decreased white matter volume and enlarged brain ventricles and extra-axial space (Fig. 1e). Electroencephalography revealed multifocal spike discharges. Conventional cytogenetic studies, array comparative genomic hybridization, and investigations for metabolic abnormalities yielded normal results.

**Fig. 1:**
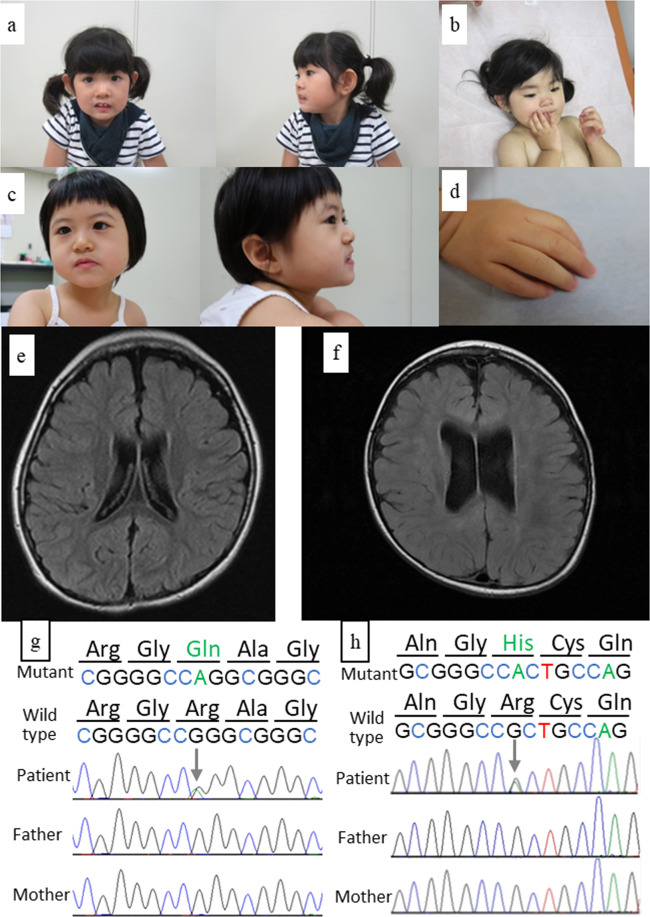
Patients with *DHX30* variants and their MRI findings. **a** Patient 2 at 3 years of age. The patient exhibited thick low-set ears, long eyelashes, interdental space, high arched palate, micrognathia, **b** tapered fingers and hand licking. **c** Patient 3 at 5 years of age. The patient exhibited internal strabismus, epicanthus, thick eye brow, long eyelashes, and **d** tapered fingers. **e** Axial T1-weighted brain MRI of Patient 2 demonstrated a decreased white matter volume and enlarged brain ventricles and extra-axial space. **f** Axial T2-weighted fluid-attenuated inversion recovery brain MRI of Patient 3 demonstrated significant decreases in the volume of the midbrain and ventricular dilatation and completed myelination with remaining multiple areas of patchy increased T2 signal around the cerebral white matter. *DHX30* variants were identified in patients 1 (**g**) and 2 (**h**) by the Sanger method. Arrows indicate variations.

Patient 3 was a 5-year-old girl who was the second child of healthy and nonconsanguineous Japanese parents. She had an unaffected older sister. Her birth weight was 2556 g (−0.9 SD), length was 46 cm (−1.1 SD), and head circumference was 32.4 cm (−0.5 SD) at 38 weeks of gestation. She suckled at the breast well. However, she could not eat well after starting baby food. Delayed neck control was noted at 5 months of age. She was referred to a city hospital at 11 months of age, and an MRI brain scan indicated brain atrophy. She could roll over at 7 months of age, control her head at 12 months of age, and sit at 24 months of age. She was diagnosed by whole exome sequencing (WES) in the United States of America, where she moved at 26 months of age. After she returned to Japan, she visited our center at 5 years of age. Her height was 95.1 cm (−3.1 SD), weight was 10.9 kg (−3.1 SD), and head circumference was 46.4 cm (−2.4 SD). She showed muscular hypotonia and moved only by rolling over. She had feeding difficulties. Her pathognomonic behaviors were hand-licking, bruxism, a happy demeanor, such as frequent smiling and laughter, and a low frustration tolerance. Physical examination revealed dysmorphic features, including internal strabismus, epicanthus, thick eyebrows and long eyelashes, and a high arched palate, tapered fingers, joint hypermobility, and overlapping toes (Fig. 1c, d). She suffered from sleep disorder. She did not experience seizures, although electroencephalography revealed abnormalities in the awake and sleep states due to mild slowing and disorganization for her age. Brain MRI at 3 years of age revealed significant decreases in the volume of the midbrain and ventricular dilatation and arachnoid cysts in the middle cranial fossa; in addition, it showed completed myelination with remaining multiple areas of patchy increased T2 signal around the cerebral white matter (Fig. 1f). Swallowing videofluorography, electromyography, and nerve conduction velocity were normal.

With the approval of our institutional ethics committees, blood samples were collected from each participating individual, and after genomic DNA was extracted, the patients underwent WES. The pathogenicity of the variants was evaluated according to the American College of Medical Genetics and Genomics (ACMG) variant interpretation guidelines. In patient 1, a de novo missense variant in *DHX30* (NM_138615.2), c.2345 G>A, p.(Arg782Gln), was revealed (Fig. 1g). The variant was novel, although it was located just next to c.2344 C>T, p.(Arg782Trp), which was reported by Lessel et al.^2^. This variant was judged to be likely pathogenic according to the ACMG guidelines. The variant was predicted to be likely pathogenic by a SIFT score of 0, PolyPhen2 score of 1, and CADD score of 33. In patient 2, a de novo missense variant in *DHX30*, c.2354 G>A, p.(Arg785His), was revealed (Fig. 1h). This variant was previously reported by Lessel et al.^2^. In patient 3, a de novo missense variant in *DHX30*, c.2353 C>T, p.(Arg785Cys), which was also reported by Lessel et al.^2^, was revealed. All of the variants in our patients were in motif VI of the helicase core region of *DHX30*, the nucleotide-interacting motif. All protein variants in ATP-binding motif VI showed obviously reduced ATPase activities, which provided strong evidence for the pathogenicity of NEDMIAL^2^. Our findings could add to this evidence.

We reported three patients with NEDMIAL. Patient 1 is the first adult patient to be reported based on diagnosis with genetic testing for the first time in adulthood, even though he has been affected with NEDMIAL since early childhood, and he had a novel variant in *DHX30*.

Rett syndrome-like or Angelman syndrome-like features were noted in the three patients. Rett syndrome is a neurodevelopmental disorder mostly caused by variants in *MECP2*^4^. However, variants in various other genes might lead to Rett syndrome-like phenotypes. Without WES, it is difficult to diagnose NEDMIAL. NEDMIAL should be considered in the differential diagnosis of Rett syndrome and Angelman syndrome.

Brain MRI anomalies, including delayed myelination, cerebellar atrophy, dilated ventricles, cortical atrophy, and corpus callosal abnormalities, have been reported^2^. Our patients showed similar conditions. DHX30 is a member of the DEAH-box family of RNA helicases, and it is considered to be involved in several phases of the RNA lifecycle, leading to the initiation of mRNA translation^5^. Decreases in the functional level of DHX30 due to variants may lead to the progression of early developmental disorders^6^. DHX30 might play important roles in cerebral maturation, and brain MRI appears useful for the diagnosis of NEDMIAL.

In conclusion, we report a Japanese adult and two girls with NEDMIAL caused by missense variants in *DHX30*. We suggest that NEDMIAL is a novel neurodevelopmental disorder that may mimic Rett syndrome or Angelman syndrome in its early stage, and brain MRI anomalies are pathognomonic findings for the diagnosis of NEDMIAL (Table 1).

**Table 1:** Clinical characteristics of our patients and the previously reported patients with *DHX30* alterations.

Clinical findings	Patient 1	Patient 2	Patient 3	14 previously reported patients (Lessel et al.^2^, Cross et al.^3^)
Sex	Male	Female	Female	6 males, 8 females
Age at last examination	22 years	7 years	5 years	3–17 years, 6 months, 15 months
Intellectual disability	+	+	+	14/14
Speech ability	Non-verbal	Non-verbal	Non-verbal	20 words, 4 words, 11 non-verbal, 1 N/A
Motor development delay	+	+	+	14/14
Muscular hypotonia	+	+	+	14/14
Age at walking (years)	−	−	−	2 8/12, 6, 3, 5, 8, 11 no walking, 1 N/A
Gait abnormalities	No independent walking	No independent walking	No independent walking	6 ataxic, 6 no independent walking, 2 N/A
Autistic features	+	+	+	+(7/12), 2 N/A
No purposeful movement of hands	+	+	+	+(5/12), 2 N/A
Bruxism	−	+	+	+(1/12), 2 N/A
Sleep disturbance	+	−	+	+(7/14)
Seizure	−	+	+	+(3/14)
Feeding difficulties	−	−	+	+(11/14)
Strabismus	−	−	+	+(6/14)
Joint hypermobility	+	−	+	+(6/14)
Unilateral cryptorchidism	−	N/A	N/A	+(4/6)
Brain MRI anomalies	+	+	+	+(10/14)
*DHX30* alteration	c.2345 G>A, p.(Arg782Gln)	c.2354 G>A, p.(Arg785His)	c.2353 C>T, p.(Arg785Cys)	c.1478 G>A, p.(Arg493His) (2/14), c.1685A>G, p.(His562Arg) (1/14), c.2093 C>T, p.(Ser698Phe) (2/14) c.2342 G>A, p.(Gly781Asp) (2/14), c.2344 C>T, p.(Arg782Trp) (3/14), c.2353 C>T, p.(Arg785Cys) (3/14), c.2354 G>A, p.(Arg785His) (1/14)

*+* present, *−* absent, *N/A* not applicable.

## HGV database

The relevant data from this Data Report are hosted at the Human Genome Variation Database at 10.6084/m9.figshare.hgv.3024; 10.6084/m9.figshare.hgv.3027; 10.6084/m9.figshare.hgv.3030.
